# Heterologous Expression of *OtsB* Increases Tuber Yield and Phenotypic Stability in Potato under Both Abiotic and Biotic Stresses

**DOI:** 10.3390/plants12193394

**Published:** 2023-09-26

**Authors:** Britany Lauren Morgan, Tayebeh Kakeshpour, Alessandro Occhialini, Gabriella King, Megan Sichterman, Stacee A. Harbison, Stephen B. Rigoulot, Holly Brabazon, Charles Neal Stewart, Scott C. Lenaghan

**Affiliations:** 1 Center for Agricultural Synthetic Biology, University of Tennessee, Knoxville, TN 37996, USA; britanylmorgan@gmail.com (B.L.M.); aocchial@utk.edu (A.O.); msichter@vols.utk.edu (M.S.); ssnyde16@utk.edu (S.A.H.); hbrabazo@utk.edu (H.B.); 2 Department of Plant Sciences, University of Tennessee, Knoxville, TN 37996, USA; tayebeh.kak@gmail.com (T.K.); gking11@vols.utk.edu (G.K.); 3 Syngenta Crop Protection, LLC, 9 Davis Drive, Research Triangle Park, NC 27709, USA; 4 Department of Entomology and Plant Pathology, University of Tennessee, Knoxville, TN 37996, USA; 5 Department of Food Science, University of Tennessee, Knoxville, TN 37996, USA

**Keywords:** sustainable agriculture, *Solanum tuberosum* var. ‘Desiree’ (potato), *trehalose-6-phosphate phosphatase* (*OtsB*), biotic stress, abiotic stress, competition, herbivory

## Abstract

Climate-smart and sustainable crops are needed for the future. Engineering crops for tolerance of both abiotic and biotic stress is one approach. The accumulation of trehalose, controlled through *trehalose-6-phosphate synthase* (*TPS*) or *OtsA* and *trehalose-6-phosphate phosphatase* (*TPP*) or *OtsB* genes in microbes, is known to provide protection for many microbial and fungal species against abiotic stress. The effect of trehalose accumulation in plant species is less understood. Here, we studied the heterologous expression of *Escherichia coli OtsB* in potato (*Solanum tuberosum* var. ‘Desiree’) with regards to stress tolerance. The performance of transgenic lines was assessed in both growth chambers and greenhouse mesocosms. Overexpressing potato *OtsB* lines significantly increased resilience to heat, photoperiod, herbivory, and competition when compared with wildtype plants. Most strikingly, when subjected to high temperatures, transgenic lines exhibited a significantly lower reduction in tuber yield ranging from 40% to 77%, while wildtype plants experienced a 95% decrease in tuber yield. When exposed to competitors in a selected *StSP3D::OtsB* line, tuber yield was 1.6 times higher than wildtype. Furthermore, transgenic lines performed significantly better under low-nutrient regimes: under competition, yield increased by 1.5-fold. Together, these results demonstrate that increased trehalose has the potential to create more resistant and stable crop plants.

## 1. Introduction

By the year 2050, the world population is projected to surpass 9.7 billion [1], doubling global demand for crop production. In addition to the increased burden from a rapidly growing population, increasing global temperatures created by climate change is posing stress on the already fragile food production ecosystem. In particular, the staple crop potato (*Solanum tuberosum*) is particularly sensitive to high temperatures [2,3,4], with current climate models predicting an 18–32% reduction in potato yield by 2050. Moreover, the narrow genetic base of cultivated potato intensifies the potential impact of heat stress [5]. Biotic stressors in the form of pests and weeds further exacerbate the problem reducing yield and decreasing productivity [6]. The continual evolutionary struggle between plants and pests is energy intensive. In this context, the synthesis of potent secondary metabolites by the plants diverts energy and sugars that could otherwise be used to produce tubers [7,8]. Similarly, competition with weeds, which often adapt much faster than crop species to abiotic stress, causes significant yield reduction [9,10,11], while also serving as a breeding ground for even more pests [9].

To combat the increasing abiotic and biotic stresses placed upon potato, climate-smart varieties were generated with increased stress tolerance. For example, numerous studies utilized the overexpression of various abiotic stress-related genes to develop thermotolerant potato varieties through the improvement of photosynthesis, activation of an antioxidant system, increased heat shock proteins (HSPs), increased proline content, increased chlorophyll content, and reduced electrolyte leakage [12,13,14,15]. In addition, genetic engineering of several plant resistance (R) genes was used to generate disease-resistant potatoes [16]. Most efforts for developing climate-smart potato varieties focused on remediating either biotic or abiotic stress, with limited approaches focused on mitigating both stressors through a generalized response.

Considering that both biotic and abiotic stress leads to the accumulation of harmful reactive oxygen species (ROS) in plants [17], in this work, we sought a strategy to remediate this generalized stress response in potato through modulation of trehalose. In plants, trehalose is minimally produced, but is important for direct ROS scavenging, as well as enhancing the activity of antioxidant enzymes [18]. Trehalose was also implicated as a signaling molecule in the plant biotic and abiotic stress responses [19]. Previous studies demonstrated that overexpression of genes in the trehalose biosynthetic pathway can significantly reduce the negative effects of abiotic stress. For example, tomato plants overexpressing a *trehalose-6-phosphate phosphatase/synthase* fusion, *TPSP*, had a significantly higher germination rate and enhanced expression of heat-responsive genes under stress [20]. In maize, overexpression of a rice *trehalose-6-phosphate phosphatase* (*TPP*) led to increased kernel set and harvest index under drought conditions [21]. Similar results were obtained in rice, peppers, and Arabidopsis, with increased trehalose leading to increased resistance to salt, chilling, and hypoxia [22,23,24]. Unfortunately, in potato, constitutive overexpression of *OtsB*, from *E. coli* led to stunted growth and other potentially detrimental phenotypes such as altered root system and leaf shape [25].

To avoid the phenotypic off-effects described above, we hypothesized that if *OtsB* were under the control of a developmental promoter, slow growth phenotypes would be avoided while endowing stress resistance in transgenic potato. As such, native potato flowering time promoters from *StSP3D* and *StSP6A* were targeted. *StSP3D* and *StSP6A* are FLOWERING LOCUS T-like paralogues involved in flowering and tuberization [26]. *StSP3D* is the flower-inducing signal that is mainly expressed in leaves and translocated to the shoot apex to initiate flowering, and its loss of function causes delayed flowering [26]. *StSP6A* is naturally expressed in leaves and translocated to tubers, initiating tuberigenesis, and its loss of function results in suppression of tuberization [27,28]. Studies demonstrated that these genes can be environmentally sensitive; for example, high temperatures cause downregulation of *StSP6A,* resulting in delayed and reduced tuber production [29], suggesting that they may be involved in the mobilization of abiotic stress responses.

The objective of this work was to evaluate the potential to develop climate-smart potato through overexpression of *E. coli OtsB* under the control of plant developmental promoters. *OtsB* overexpressing lines were grown with a short photoperiod, at increased temperature, and with competition under simulated field conditions with or without nutrient stress. The phenotype of the engineered lines was compared to wildtype lines under each of these scenarios to determine the performance of the lines with the goal of demonstrating a more stress-tolerant potato to address future production needs.

## 2. Results

### 2.1. Transgenic Expression of OtsB

Ten independent *OtsB* callus lines for each promoter (*StSP3D* and *StSP6A*) were transferred to selection media containing antibiotics. From those calli, the five largest plantlets demonstrating normal morphology and vigor were grown in triplicate on potting mix. Leaf discs from 3 youngest leaves were collected for DNA extraction and PCR was performed to confirm transgene presence (Supplemental Figure S1). Once plantlets established roots, they were planted into soil in duplicate and grown for 4 weeks. Leaf discs from the three youngest leaves were collected for DNA extraction. These lines were characterized by Southern blot analysis (Supplemental Figure S1). All ten lines were confirmed to contain the *OtsB* transgene and varied from single to quadruple insertions (Supplemental Figure S1). The transgenic *StSP3D::OtsB* line exhibiting the highest *OtsB* expression with a single insertion site was chosen to test in mesocosm experiments.

### 2.2. Effect of OtsB on Abiotic Stress Response

Overexpression of OtsB dampened negative effects of heat and short photoperiod on some plant traits and tuber yield while introducing positive effects of heat on others. Overall, abiotic stress treatments significantly affected all harvest plant traits except for the number of tubers (Table 1a,b, Supplemental Figure S2). In addition, transgenic lines responded to treatments differently from wildtype potato, as demonstrated by a significant genotype x treatment interaction (Table 1a,b).

**Figure 1:**
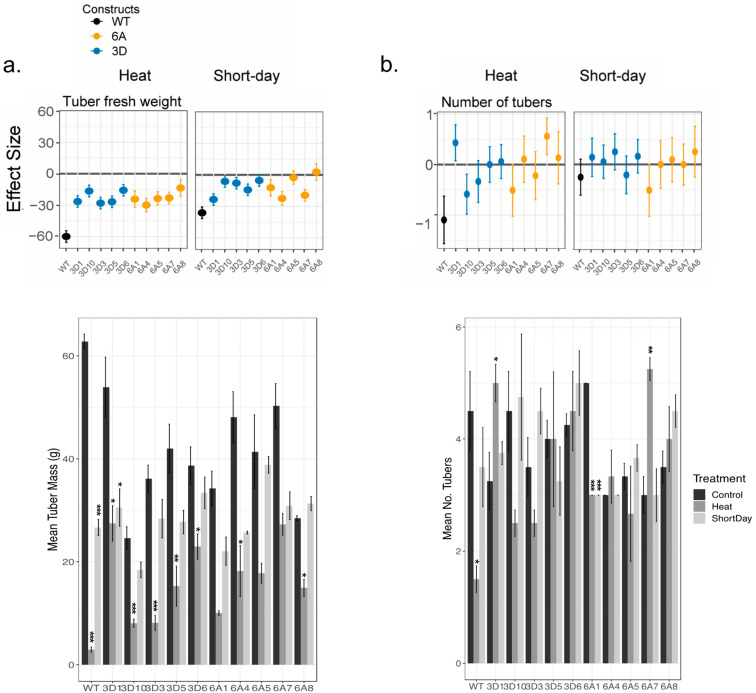
Effect of heat and photoperiod on *OtsB* transgenic constructs for belowground traits. Above: The effect size of heat and short days are plotted for (**a**) tuber fresh weight and (**b**) number of tubers. Columns represent treatment with heat on the left and short days on the right; each panel within those columns demonstrate estimates of the effect of that treatment on the specified trait. Each genotype’s estimate of effect and standard error, based on estimated marginal means, are plotted. Control of each genotype is the reference for each pairwise comparison. Color represents construct; see the key. The *x*-axis value of 0 is bolded in each graph. A value overlapping 0 indicates that there is no significant difference in traits between those grown in control vs. treatment. A positive effect indicates that individuals grown under that treatment had higher trait values, while a negative effect indicates that control individuals had higher trait vales. See Supplemental Table S2 for significant effects. Bottom: The mean and standard error of (**a**) tuber fresh mass and (**b**) the number of tubers for the same individuals. Significant effects, calculated via Dunnett’s test, of treatment within each genotype are demonstrated with the following: * *p* < 0.05; ** *p* < 0.01; and *** *p* < 0.001.

**Figure 2:**
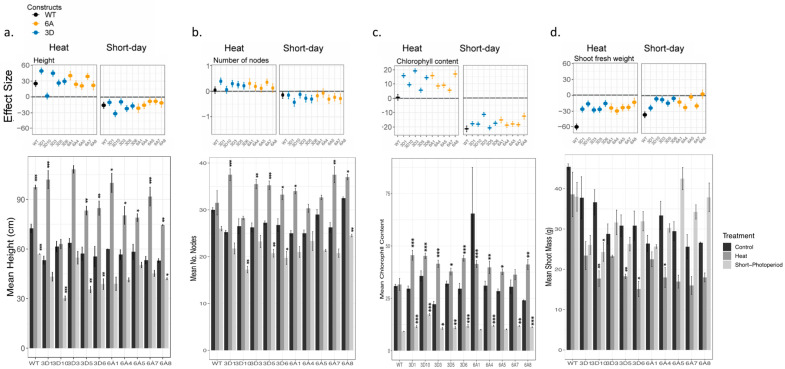
Effect of heat and photoperiod on *OtsB* transgenic constructs for aboveground traits. Top: The effect sizes of heat and short-days are plotted for (**a**) height, (**b**) number of nodes, (**c**) chlorophyll content, and (**d**) shoot fresh weight. Columns represent treatment with heat on the left and short days on the right; each panel within those columns demonstrates the estimate of effect of that treatment on the specified trait. Each genotype’s estimate of effect and standard error, based on estimated marginal means, are plotted. Control of each genotype is the reference for each pairwise comparison. Color represents construct; see the key. The *x*-axis value of 0 is bolded in each graph. A value overlapping 0 indicates there is no significant difference in traits between those grown in control vs. treatment. A positive effect indicates that individuals grown under that treatment had higher trait values, while a negative effect indicates that control individuals had higher trait vales. See Supplemental Table S2 for significant effects. Bottom: The mean and standard error of (**a**) height, (**b**) number of nodes, (**c**) chlorophyll content, and (**d**) shoot fresh weight for the same individuals. Significant effects, calculated via Dunnett’s test, of treatment within each genotype are demonstrated with the following: * *p* < 0.05; ** *p* < 0.01; and *** *p* < 0.001.

Wildtype plants suffered a substantial 95% reduction in tuber yield, from 62.8 g to 2.95 g, under heat stress, while the transgenic lines from both constructs showed higher tuber yields than the wildtype plants under heat. Despite a reduction in yield ranging from 40% to 77%, the transgenic lines still exhibited better performance under the same heat stress conditions (Figure 1a). Under short days, however, *StSP6A::OtsB* lines exhibited a trend of larger tubers than *StSP3D::OtsB* lines (Figure 1a,b). All genotypes, except for *StSP3D::OtsB* Line 10, exhibited a significant increase in plant height when grown under heat stress; plants showed up to a 95% increased growth in height compared to the control group, indicating a positive effect of heat on plant height (Figure 2a). Shorter days resulted in shorter plant stature, with the *StSP3D::OtsB* Line 10 genotype being the most affected, decreasing in stature from 61.5 cm to 30.25 cm (Figure 2a).

While the number of tubers was not affected by either heat or short days for any genotype, transgenic lines varied in whether heat or short days affected the number of nodes on aboveground biomass. When exposed to heat stress, *StSP3D::OtsB* Lines 1 and 3, as well as StSP6A::OtsB Line 7, increased their node numbers significantly up to 48.5% (Figure 2b). Shorter days caused *StSP3D::OtsB* Line 10 to significantly reduce its node number by 32% (Figure 2b).

Transgenic lines varied in whether they demonstrated a stronger reduction in chlorophyll content after exposure to short days when compared to wildtype, but all transgenic lines had significantly higher chlorophyll content when exposed to heat (Figure 2c). *StSP3D::OtsB* Lines 3 and 6 and *StSP6A::OtsB* Lines 1 and 8 experienced the least difference in chlorophyll content between control and short-day treatments (Figure 2c). While wildtype had no significant effect of heat on chlorophyll content of leaves, all transgenic lines of *both StSP3D::OtsB* and *StSP6A::OtsB* demonstrated increased chlorophyll content when grown under heat stress (Figure 2c).

### 2.3. Effects of OtsB Gene Expression on Biotic Stress

**Figure 3:**
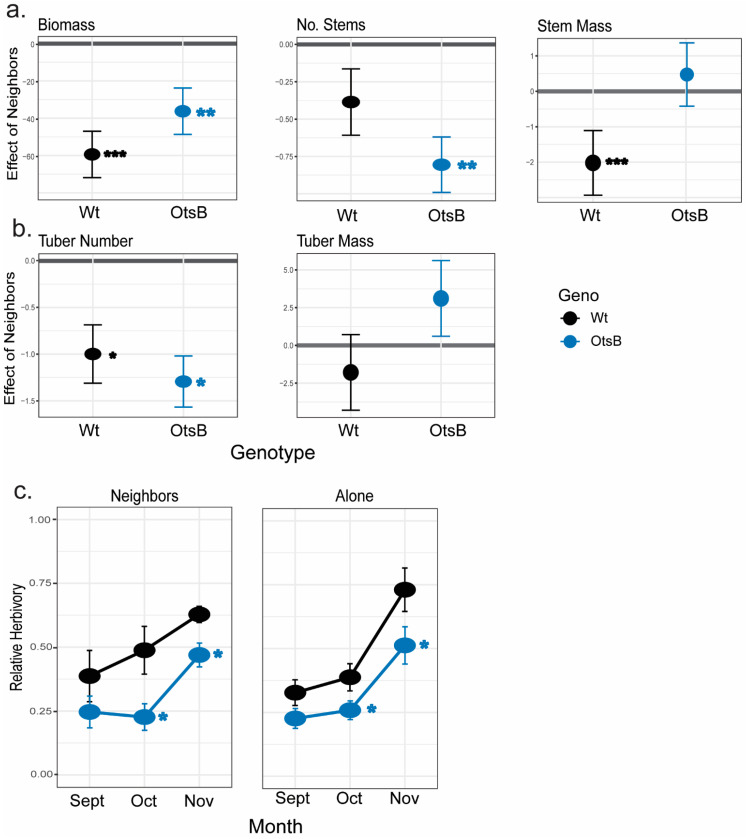
Effect of competition and herbivory on wildtype and *SP3D::OtsB* transgenic line 10 in mesocosm 1. The effect size of competition is plotted for (**a**) aboveground traits of biomass, number of stems, and stem weight and (**b**) belowground traits of tuber number and weight. Each genotype’s estimate of effect and standard error, based on estimated marginal means, are plotted (**a**,**b**). Individuals grown alone are the reference for each pairwise comparison of each genotype. Color represents genotype; see the key. The *x*-axis value of 0 is bolded in each graph; a negative effect indicates that individuals grown alone had higher trait values. The mean relative herbivory and its standard error (**c**) are plotted for each month. Significant difference of transgenic line from wild-type for relative herbivory is demonstrated with an asterisk (*p* < 0.05), from ANOVA based on GLM. See Supplemental Table S3 for significant effects. Significant chi-square values are bolded: *** *p* < 0.001, ** *p* < 0.01, and * *p* < 0.05.

Overexpression of *OtsB* led to more phenotypic stability for some aboveground traits when exposed to neighbors, while also introducing a positive effect of competition on tuber yield and a negative effect on stem mass. *StSP3D::OtsB* Line 10 had lower biomass than wildtype when grown alone, averaging 56 g compared to 69 g, but under competition with neighbors, Line 10 had greater aboveground biomass than wildtype, with 20.5 g compared to 14.9 g, respectively (Figure 3a, Table 2, Supplemental Figure S2). When exposed to competition, the transgenic line had heavier stems than wildtype (Figure 3a, Table 2). While wildtype exhibited no significant difference in stem number between competitive and non-competitive mesocosms (6.8 and 10, respectively), the transgenic line had significantly fewer stems when grown under competition than when grown alone (7.4 and 18, respectively) (Figure 3a). Wildtype plants growing under competitive conditions produced significantly lower tuber mass than when grown alone, 12.5 g and 60.2 g, respectively; however, the tuber yield for the transgenic line was unaffected by competition with neighbors (31.2 g vs. 20.2 g) (Figure 3b). Both genotypes produced significantly fewer tubers when grown under competition (Figure 3b). Together, these results demonstrate that increased OtsB expression caused plants to grow larger with larger tubers when exposed to competition when compared with wildtype (Supplemental Figure S2).

*OtsB* overexpression reduced relative herbivory regardless of community. *StSP3D::OtsB* Line 10 demonstrated reduced relative herbivory when compared to wildtype, by ~1.3×–1.5× lower herbivory (Figure 3c). Relative herbivory increased across time for both genotypes; however, the transgenic line had less relative herbivory across the months of this experiment (Figure 3c). There was no significant difference in relative herbivory between community types for either genotype.

**Figure 4:**
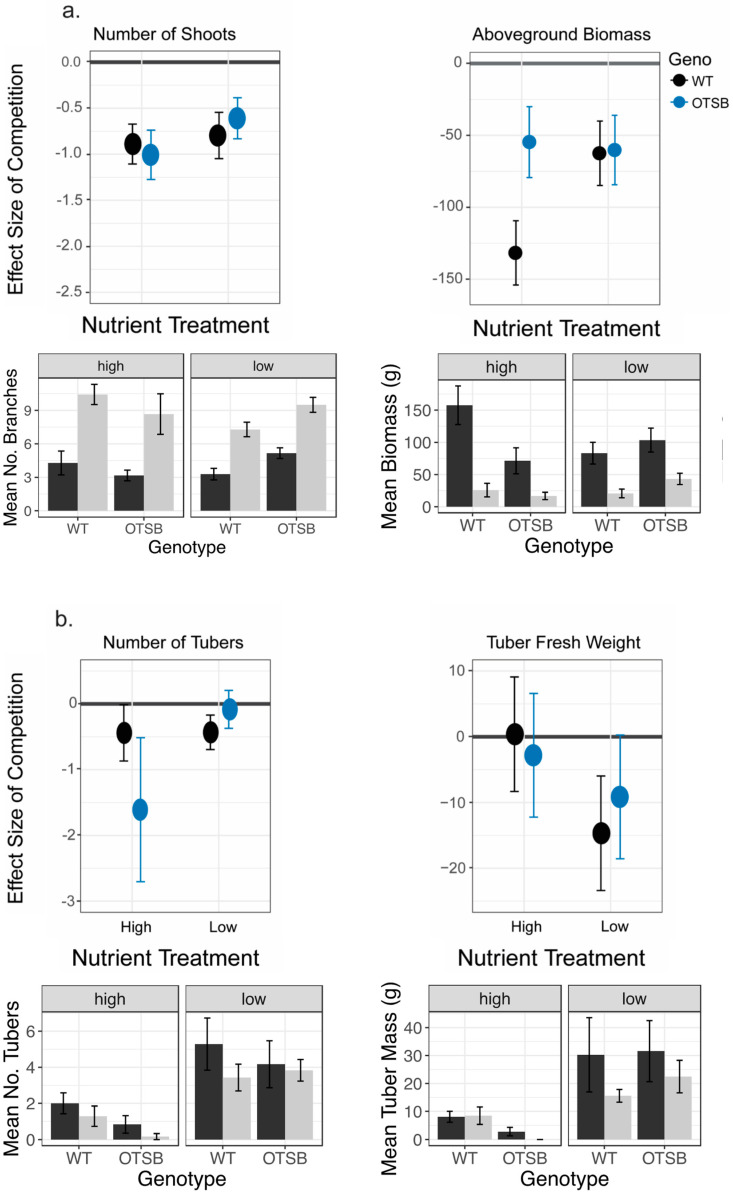
Effect of competition in mesocosm 2 under high and low nutrient availability. Top: The effect size of competition is plotted for (**a**) aboveground traits of number of stems and biomass and (**b**) belowground traits of tuber number and weight. Nutrient regime is demonstrated on the *x*-axis. Each genotype’s estimate of effect and standard error, based on estimated marginal means, are plotted. Individuals grown alone within each nutrient regime are the reference for each pairwise comparison of each genotype. Color represents genotype; see the key. The *x*-axis value of 0 is bolded in each graph; a negative effect indicates that individuals grown alone had higher trait values. See Supplemental Table S3 for significant effects. Bottom: the mean and standard efforr of the (**a**) aboveground and (**b**) belowground traits for the same individuals with those grown alone in dark gray and those grown under competition in light gray.

*OtsB* overexpression dampened the effect of neighbors on biomass under high nutrient availability and tuber yield when under low nutrient availability in mesocosm 2. The trend of genotypic differences in response to competition demonstrated a significant interaction with the nutrient regime (Supplemental Figure S3). Under low nutrient conditions, plants grown under competition developed significantly lower aboveground biomass and fewer shoots for both genotypes (Figure 4a, Table 3). For wildtype, plants grown under high-nutrient regimes exhibited a 6-fold reduction in biomass when exposed to competition, and those grown under competitive conditions in low nutrient regimes exhibited a 3.2-fold reduction in biomass. When plants were grown without competition under low nutrient conditions, *StSP3D::OtsB* Line 10 exhibited larger aboveground biomass than wildtype (~101 g vs. ~78 g), but when grown in the high-nutrient regime, wildtype plants grown alone demonstrated the highest biomass (~151 g) (Figure 4a). *StSP3D::OtsB* Line 10 was not significantly different from wildtype under either nutrient regime regarding the number of shoots (Figure 4a, Table 3). Neither genotype demonstrated a significant effect of competition on the number of tubers under either nutrient regime (Figure 4b). For tuber fresh weight, there was no significant effect of competition under high-nutrient conditions for either genotype, but when plants were grown under low nutrient conditions, wildtype exhibited a significant reduction in tuber mass by about 50% when grown with neighbors, while *StSP3D::OtsB* Line 10 did not (about 20% reduction) (Figure 4b). These results demonstrate that nutrient regime influenced the magnitude of the effect of competition, and increased *OtsB* expression affected response to competition in a manner that interacted with nutrient availability.

*OtsB* overexpression eliminated the positive effects of supplemental nutrients. Plants demonstrated a negative effect of growing under high nutrient availability on belowground traits for both genotypes, while demonstrating positive effects on aboveground traits in wildtype only (Table 4). High nutrient availability resulted in significantly decreased tuber number for both genotypes regardless of community type (Figure 4b, Supplemental Figure S4). Wildtype plants exhibited significant reductions in tuber fresh weight under high nutrient availability when grown alone (30 g vs. 8.9 g, low vs. high nutrient availability, respectively), while the effect of high nutrient availability on tuber fresh weight was lost when plants were grown under competitive conditions (15 g vs. 9.0 g, low vs. high nutrient availability, respectively) (Supplemental Figure S4). For *StSP3D::OtsB* Line 10, however, the significant reduction in tuber fresh weight when exposed to high nutrient availability persisted regardless of community type, and tuber yield was lowest for Line 10 plants grown under competition in high-nutrient regimes (~1.5 g) (Supplemental Figure S4). For shoot number, there was no significant effect of nutrient regime for either genotype in any community (Table 4). Wild-type plants grew significantly larger under high nutrient availability when grown alone, but this positive effect of nutrient regime was lost when wildtype was grown with competitors (Table 4). *StSP3D::OtsB* Line 10, however, demonstrated no significant difference in biomass when exposed to high nutrient availability regardless of community (Table 4). Together, these results suggest that potato performs best under lower nutrient availability and increased OtsB decreases the need for supplemental nutrients.

### 2.4. Increased OtsB Expression Altered Expression of Other Stress Related Genes

**Figure 5:**
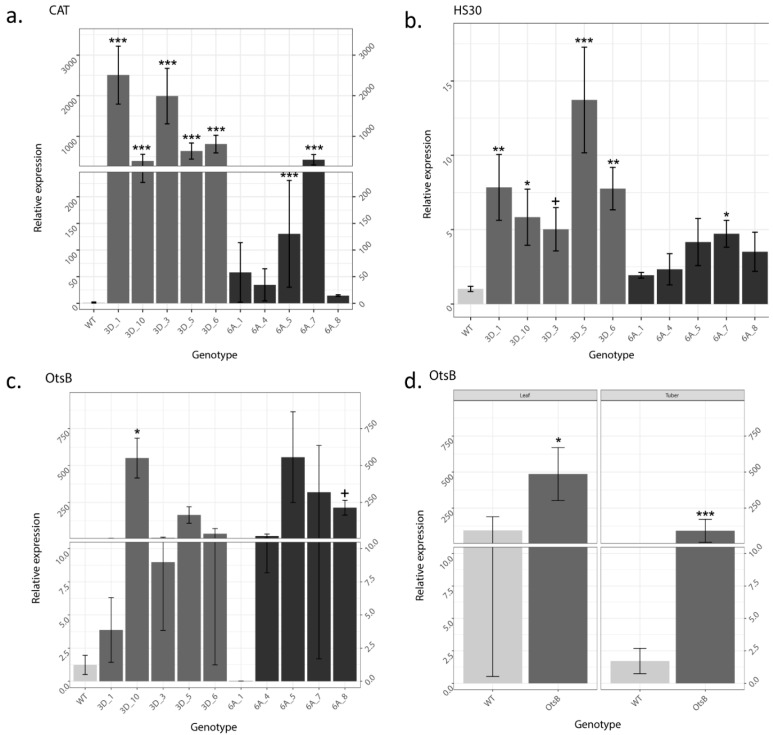
Comparison of transcript levels between wildtype and *OtsB* transgenic lines under control conditions in growth chambers; (**a**) *catalase* (*CAT*); (**b**) *heat shock protein 30* (*HS30*); (**c**) *OtsB*; and mesocosm 1 without neighbors (**d**) *OtsB*. Leaf tissue relative expression is shown in all four panels, and (**d**) includes relative expression from harvested tubers. Data are analyzed using 2^−ddCt^. Means were compared using Dunnett’s test, + *p* = 0.05, * *p* < 0.05; ** *p* < 0.01; and *** *p* < 0.001. Error bars represent ±SE of three biological replicates.

Both *StSP3D::OtsB* and *StSP6A::OtsB* transgenic lines showed increased expression of *CAT* and *HS30* compared to wildtype potato (Figure 5a,b). Transgenic lines of both *StSP3D::OtsB* and *StSP6A::OtsB* had up to ~2500 and ~400 times increased *CAT* expression, respectively. All five lines of *StSP3D::OtsB* had significantly higher relative expression when compared to wildtype under control conditions. However, *StSP6A::OtsB* exhibited greater inter-line variability, with significantly higher relative expression in Lines 4 and 7. Expression of *HS30* showed up to ~14 and ~5-fold induction in transgenic lines of both *StSP3D::OtsB* and *StSP6A::OtsB*, respectively. All five *StSP3D::OtsB* lines demonstrated significantly higher expression than wildtype under control conditions; while all *StSP6A::OtsB* lines exhibited a trend of higher expression than wildtype, Line 7 was the only line with a significant increase in expression. Under control conditions, total *OtsB* transcripts (native and transgenic expression) varied greatly between lines. Only *StSP3D::OtsB* Line 10 and *StSP6A::OtsB* Line 8 expressed total *OtsB* at significantly higher levels than wildtype (Figure 5c), suggesting that transgenic *OtsB* may interact with native expression of *OtsB* depending on insertion site. Mesocosm 1 plants demonstrated significantly higher total *OtsB* expression in both leaves and tubers when grown alone (Figure 5d). Expression of *OtsB* increased in tubers of both wildtype and *StSP3D::OtsB* Line 10 plants in the presence of neighbors, while in leaves, *OtsB* expression decreased in wildtype and remained the same in *StSP3D::OtsB* Line 10 (Figure 5d). These results suggest that the increased tuber yield observed under both biotic and abiotic stress conditions might be due to increased expression of antioxidant enzymes.

## 3. Discussion

### 3.1. Exogenous Expression of OtsB Led to Reduced Negative Effects of Stress and Higher Yield

In the current study, transgenic plants with increased trehalose demonstrated increased phenotypic stability through significant reductions in the effect size of heat, photoperiod, and biotic stresses. However, while the trends of response to stress were very strong, the independent lines demonstrated a large degree of variability. In most plant species, the presence of trehalose in tissues is extremely low, with the exception of resurrection plants. The transgenic lines generated in this work varied in copy number from one to four (Supplemental Figure S1). When testing tobacco for overexpressed *E. coli* trehalose genes, transgenic plants demonstrated a decreased response to drought stress. These plants accumulated up to 0.2 mg/g trehalose in their leaves [30]; in comparison, resurrection plants have up to 19 mg/g in their leaves [31]. The variation in plant response seen in our experiments could be driven by variation in the location of gene insert, driving gene expression to cause variable trehalose accumulation in plant tissues. Furthermore, the triple insertions, *StSP3D::OtsB* Line 3 and *StSP6A::OtsB* Line 8, demonstrate the weakest overall phenotypes in control conditions, which suggests that hyperaccumulation of trehalose may be associated with a fitness penalty.

Throughout the breadth of life, trehalose is demonstrated to increase tolerance to a variety of abiotic stressors [32,33]. In microbes, trehalose produced from *OtsA/B* genes was demonstrated to protect membranes and resistance against increased temperature and salinity [34,35,36,37]. The accumulation of trehalose led to increased thermotolerance in several species of fungi, while it appeared to have limited protection against water limitation [38,39,40]. In plant studies of trehalose and related sugars, abiotic stresses such as heat, cold, salt, and drought are focal stimuli [19,41]. In several plant species, from Arabidopsis to rice, increased trehalose led to increased cold, salt, and drought tolerance [19,30,41,42]. However, as these studies failed to test the effects of increased trehalose throughout the entire life cycle and/or grow plants in controlled growth chambers, they lacked the realistic simulation of natural conditions experienced by crop plants and how it affects their yield. In addition to abiotic stress, trehalose was implicated in affecting certain species interactions. In microbes and fungi, trehalose accumulation can alter microbial behavior and phenotype to increase virulence [36,43,44,45,46]. Trehalose exposure in plants can mobilize immune responses [47,48], suggesting a complicated balance in the role of trehalose in the arms race between plants and their pathogens.

Furthermore, results from our first mesocosm experiment demonstrate reduced relative herbivory in the *StSP3D::OtsB* transgenic line when compared to wildtype plants. The metabolism and production of trehalose was found to influence plant defense response to phloem-feeding insects in Arabidopsis and tomato [49,50,51]. In addition, as trehalose accumulation can lead to altered phenotypes including thicker leaves, increased branching, and altered leaf shape [30,41,52], insects may face difficulty feeding compared to wildtype plants, which in turn may reduce the relative herbivory of transgenic plants. Tomato, tobacco, wild sugar cane (*Saccharum spontaneum*), and Arabidopsis were shorter when accumulating trehalose through either overexpression of trehalose synthesis genes or through knockdowns of trehalose catalysis genes [30,53,54,55,56]. Furthermore, as changes in trehalose concentrations can impact the sugar and starch content of plant tissues [54,57,58], insects may be less attracted to transgenic plants. Alterations in sugar concentrations of plant tissues can also affect the creation of volatile organic compound (VOC) precursors [59,60], suggesting that the accumulation of certain sugars and related hormones may affect the attraction of insects through altered VOC profiles. However, as our experiments did not include the collection or analysis of volatiles, we are unable to directly test whether this plant–insect interaction was mediated by volatiles. Further experiments using insects can test whether attraction is different between trehalose-accumulating plants compared to wildtypes driven by volatile emission; or alternatively, whether attraction is similar between plants, but other phenotypes protect trehalose-accumulating plants from herbivory.

Another important biotic stressor for plants is competition. These negative plant–plant interactions require prioritizing removing “weeds” from around focal plants. For most crop production, this process is completed using herbicides that are often dangerous for humans and ecosystems surrounding farmlands [61,62,63]. Therefore, reducing the negative effects of competition from weedy neighbors in crops can minimize or eliminate herbicide use at the benefit of human and ecosystem health alike. In our experiments, we demonstrated that wildtype plants suffer greater negative effects of competition under simulated field conditions than transgenic *StSP3D::OtsB* plants, and in mesocosm 1, neighbors even introduced a positive trend on tuber yield. To the authors’ knowledge, our experiments were the first to examine the effect of trehalose on plant competition. However, responses to cues of competition are well defined. One well-studied cue of neighbor presence is through the detection of alterations in light quality and quantity. Plants alter their morphology and phenology, or life history transitions, when exposed to reduced red: far-red light and/or total irradiance [64,65]. The coordination of these changes in phenotype, also known as the “shade avoidance syndrome” or “shade avoidance response”, are mediated through several key phytohormones through the regulation of phytochromes [65,66,67,68,69]. Trehalose was shown to interact and interfere with biosynthesis of phytohormones. For example, one plant hormone regulated via shade avoidance, auxin, was found to act downstream of trehalose in seed filling of garden peas (*Pisum sativum*) [58]. Exogenous application of trehalose to Arabidopsis seedlings led to the repression of several nitrilase genes, which can regulate auxin metabolism [42]. In sugar cane, plants with increased expression of trehalose-related genes and expression of plant hormone transduction pathways demonstrated the highest enrichment when compared with genotypes with lower expression of trehalose-related genes [54]. Therefore, plants with increased accumulation of trehalose likely experience altered basal hormone levels, which may reduce the downstream induction of negative responses to competition. We closely monitored phenotypic stability in our transgenic potato lines. While some variations in growth were observed, there were no consistent or significant phenotypic aberrations related to auxin metabolism. Overall, the transgenic lines exhibited healthy growth patterns and phenotypic stability under normal and stress conditions, suggesting that heterologous *OtsB* gene expression did not appear to disrupt auxin metabolism.

Beyond responding to shade, growth habit and morphology can affect the strength of competition between plants. For example, longer branches and larger leaves led to increased competitive ability in pea plants (*Pisum sativum*) [70], and in *Ranunculus reptans,* increased stolon length and number of rosettes increased fitness under competition [71]. *Impatiens capensis* demonstrated altered meristem allocation of traits with neighbors [72]. Transgenic plants in our experiments varied in phenotypes but demonstrated a strong trend of reduced stature and shoot fresh weight when compared to wildtype potato plants. When grown under competition, these phenotypes conferred a competitive advantage. Furthermore, auxin was demonstrated to regulate branching number in Arabidopsis [73], suggesting that alterations in morphology present in trehalose-accumulating plants may be due to interactions between trehalose and plant hormones. Alternatively, transgenic plants may experience a synergistic interaction between hormone biosynthesis and beneficial phenotypes in regard to plant competition. Together, the transgenic plants grown here demonstrated reduced negative effects of competition and stable yield, which may reduce the efforts of herbicide use when planted in agricultural fields.

### 3.2. Increased OtsB Expression Has Potential to Create a Better Climate-Smart Crop

A major concern for climate change and agricultural production is the use of fertilizer. Overuse of fertilizer can contribute to climate change through the release of emissions [74,75]. In addition to its direct effect on climate change, the overuse of fertilizer has other detrimental environmental effects, such as algal blooms and subsequent anoxic zones from run-off [76,77]. Current crop production utilizes large quantities of fertilizer to meet yield demands [78]. Therefore, creating crops with a lower fertilizer demand is essential for more climate-responsible agriculture. In mesocosm 2, we examined the effect of nutrient availability on fitness and response to competition. Both wildtype and the *OtsB* line demonstrated significant and strong negative effects of high-nutrient environments on plant vigor and tuber yield. The detrimental effects of over-fertilization may be caused by increasing the presence of fungal pathogens [79] or altering plant metabolism [80,81]; while we did not notice fungal disease, we cannot defiantly eliminate their presence as we did not confirm via method like PCR. Further studies that quantify the presence and pathogenicity of fungi or directly measure metabolism are required to elucidate the cause of these effects. We found the transgenic *OtsB* line demonstrated increased negative effects of competition and poorer growth under high-nutrient conditions, demonstrating a reduced nutrient demand for this line when compared to wildtype. The most likely cause is altered metabolism and relative concentrations of secondary metabolites that can affect plant growth and tuber quality. In the low nutrient mesocosms, supplemental fertilizing occurred only once at establishment of the mesocosm, and plants relied on the field soil/potting mix thereafter. Transgenic lines demonstrated reduced effects of competition when grown without supplemental fertilizer compared to those grown under high fertilizer regimes (Figure 4, Table 4). In addition, transgenic lines demonstrated reduced tuber mass when grown under high nutrient availability; therefore, these plants may eliminate the need for supplemental fertilizer and discourage their use.

Beyond the elimination of fertilizers and herbicides, transgenic plants can alter the growing season requirements for crops. In our experiments, we found transgenic lines of both constructs to demonstrate reduced negative effects of short days (Figure 1 and Figure 2, Table 2). In potato crop lines, both optimum temperature and day length are required to maximize yield. However, with increased food demand, potatoes are cultivated in different climates; therefore, reduced sensitivity to temperature and day length increases the range of suitable arable land for this crop [5]. In addition, potato cultivars demonstrate different responses to photoperiod [82]. Although short days are required for many cultivars to initiate tuberization, these conditions can also reduce photosynthesis and chlorophyll content, leading to reduced yield [82]. A balanced sink and source allocation in less-sensitive potato cultivars may increase potato yield [82]. Furthermore, as climate change continues to alter temperature, the cues of temperature and photoperiod may become decoupled. The use of a potato cultivar with reduced sensitivity to photoperiod may be necessary for human food demands in the future. In addition, photoperiod insensitivity may allow multiple growing seasons of potatoes in their current arable regions.

In summary, transgenic *OtsB* potato plants had increased tolerance to both abiotic and biotic stresses. When exposed to heat and short days, transgenic lines of both *StSP3D::OtsB* and *StSP6A::OtsB* exhibited reduced negative effects on biomass, chlorophyll content, and tuber yield. While transgenic lines produced lower tuber mass than wildtype under control growth chamber experiments, all transgenic lines produced more tuber mass than wildtype under heat stress. Our gene expression analysis revealed elevated levels of stress-related genes, including catalase and heat shock protein 30, in the transgenic lines compared to the wildtype. These findings suggest that the introduced trehalose synthesis genes may indirectly enhance tuber yield under heat stress by upregulating key stress response genes. Furthermore, the observed enhancement in tuber yield may be attributed to the protective role of trehalose in stabilizing cellular structures and its potential indirect effects on metabolic pathways and hormonal signaling involved in tuber development. When grown in mesocosm experiments with competitors, transgenic *StSP3D::OtsB* plants exhibited reduced negative effects of competition, higher yield, and lower relative herbivory than wildtype plants. In addition, transgenic *StSP3D::OtsB* plants grew better under low nutrient availability in the second mesocosm experiment. Together, these results demonstrate that transgenic *StSP3D::OtsB* and *StSP6A::OtsB* plants exhibit more stable phenotypes and higher yield when exposed to abiotic stress than wildtype. Further evaluation of *StSP3D::OtsB* plants showed that our transgenic plants also perform better under biotic stress. Through reduced fertilizing, herbicide use, and increased optimal niche breadth, transgenic *OtsB* potato plants have the potential to be engineered to create a climate-smart crop.

## 4. Materials and Methods

### 4.1. Vector Construction

**Figure 6:**
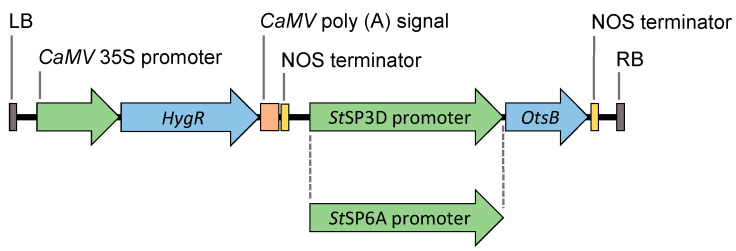
Schematic representation of *SP3D::OtsB* and *SP6A::OtsB* constructs. LB, left border T-DNA repeat; *Cauliflower mosaic virus* (*CaMV*) *35S* promoter; *HygR*, hygromycin resistance gene; *Cauliflower mosaic virus* (*CaMV*) poly (A) signal; nopaline synthase (NOS) terminator; *Solanum tuberosum* SP3D/SP6A promoter; *E. coli OtsB*; nopaline synthase (NOS) terminator; and RB, right border T-DNA repeat.

Two plant expression vectors expressing the *E. coli OtsB* gene driven by *StSP3D* or *StSP6A* promoters were constructed using the Golden Gate cloning system described by Engler [83]. We did not perform codon optimization for the *OtsB* gene in our study; it was used in its native form. This decision was influenced findings from *Populus trichocarpa* (Rigoulot, unpublished), which suggested that using the native gene could lead to alterations in plant architecture. *OtsB* from *E. coli* and *StSP3D* and *StSP6A* promoter sequences from *Solanum tuberosum* cv. ‘Desiree’ were amplified and BbsI cloning sites were added for compatibility with Golden Gate assembly. Amplified sequences of *OtsB* and promoters were cloned into pICH41308 (H2) and pICH41295 (G2) level 0 acceptors, respectively. The hygromycin resistance gene (*HygR*), which is used as a plant selectable marker, was cloned into the pICH86966 (E12) level 2 acceptor vector. *SP3D/SP6A*, *OtsB,* and nopaline synthase terminator (NOS) cassettes were cloned into the pICH86966-Hyg binary vector (Figure 6). Primer sequences are listed in Supplementary Table S2, and gene abbreviations are listed in Table 5.

### 4.2. Plant Material and Transformation

Vectors were transformed into wildtype *Solanum tuberosum* cv. ‘Desiree’, provided by UW-Madison Wisconsin Seed Potato Certification Program (WSPCP) tissue culture laboratory, using Agrobacterium-mediated transformation [84]. Stable T0 transgenic potato lines were generated and maintained on MS media (Plant Phytotec Labs, Lenexa, KS, USA) containing the antibiotic hygromycin. To verify transgene insertion, rooted plantlets were transplanted into soil in duplicate and grown for 4 weeks. Leaf disks from the three youngest leaves of each plant were collected in 2 mL tubes, quickly immersed in liquid nitrogen, and stored at −80 °C. DNA was extracted using a CTAB method modified from [85], then used in PCR. These same plants were verified via Southern blot [86] using 2 μg of genomic DNA from the two biological replicates of each line and wildtype. DNA was cut using BamHI, HaeII, and Spel-HF enzymes. Fragments were separated on a 0.9% agarose gel. DIG hybridization probes were generated using the OtsB-bbsl primer (Supplementary Table S4) with the Roche PCR DIG Probe Synthesis Kit. The probe membrane was placed on the agarose gel, UV crosslinked, hybridized, and imaged according to the Mellars and Gomez (2011) protocol (Supplementary Figure S1).

### 4.3. Plant Propagation

Focal potato plants were propagated through tissue culture under 12 h light at ~22 °C. Cuttings were grown on MS media (WT) or MS media plus hygromycin (to ensure transgenic lines maintained the gene of interest) until they reached at least 3 cm in height after about 4 weeks. Then, each cutting was trimmed to the same height of 2.5 cm and all were placed into new MS media with thiamine to help stimulate root development. Once cuttings grew primary roots of at least 2 cm in length (about 10 days later), they were transferred into potting mix (PRO-MIX BK25, Premier Tech Horticulture, Quebec, QC, Canada).

### 4.4. Abiotic Stress (Heat/Short Photoperiod)

To evaluate whether increased *OtsB* expression influenced response to abiotic stress, wildtype and five independent transgenic lines of each *StSP3D::OtsB* and *StSP6A::OtsB* genotypes were evaluated under heat and short photoperiod conditions. T0 transgenic potato cuttings prepared from tissue culture, as described above, were transferred into 4-inch plastic pots filled with potting mix. Plants were grown in growth chambers under optimal growth conditions for potato (16/8 h day/night photoperiod and 21/17 °C day/night temperatures) and watered as needed with bi-weekly fertilizer supplementation (Peters 20-20-20, J.R. Peters Inc., Allentown, PA, USA). Seedlings were allowed to grow and establish for six weeks to eliminate transplant shock effects. Seedlings were then divided and randomly assigned to control, heat, and short photoperiod groups. Six plants per line per treatment were used for each heat and short photoperiod experiment. For control treatment, growth chamber settings remained on the same day length and temperature. For heat and short photoperiod treatments, growth chamber settings were adjusted to 16/8 h day/night photoperiod and 30/26 °C day/night temperatures or 8/16 h day/night photoperiod and 21/17 °C day/night temperatures, respectively.

### 4.5. Trait Measurement

In growth chamber experiments, the effect of abiotic stress was evaluated by analyzing fresh aboveground biomass, the number of branching nodes, and the number and weight of tubers after senescence. Chlorophyll content was measured using a chlorophyll meter (Opti-sciences, CCM-200 plus) once plants were exposed to treatments for one month. An electrolyte leakage assay [87] was completed to test for membrane permeability; see Supplemental Information for details.

### 4.6. qRT-PCR

Relative expression of *OtsB* and abiotic stress-related genes including *catalase* (*CAT*) and *heat shock protein 30* (*HS30*) were measured. Primer sequences of genes of interest are listed in Supplementary Table S4. Total RNA was isolated from leaf tissue of wildtype and all transgenic lines from heat experiments. Three leaf disks from each plant were collected in 2 mL tubes, quickly immersed in liquid nitrogen, and stored at −80 °C. The Quick-RNA Miniprep Kit (Zymo Research, Irvine, CA, USA) was used to isolate total RNA. Five-hundred ng total of RNA was used to synthesize the first strand of cDNA using the ZymoScript RT PreMix Kit (Zymo Research, Irvine, CA, USA). The qRT-PCR reactions were prepared using PowerUp SYBR™ Green Master Mix (Fisher Scientific, Hampton, NH, USA). L8 or EFIα were used as reference genes to normalize Ct values. After PCR completed in clear 384-well PCR plates with four biological and two technical replicates for all genotypes, data were analyzed using the Livak method (2^−ddCt^) [88]. Means were compared using R v. 4.0.0 (R Core Team 2021), Dunnett’s test, + *p* = 0.05, * *p* < 0.05; ** *p* < 0.01; and *** *p* < 0.001.

### 4.7. Biotic Stress (Competition, Herbivory, Nutrient Flux)

#### 4.7.1. Mesocosm 1: Competition and Herbivory under Summer Temperatures

- Plant propagation

Focal potato plants were propagated through tissue culture as described above. The highest expressing transgenic line with a single insert, *StSP3D::OtsB* Line 10, was chosen for use in the mesocosm experiments (Supplementary Figure S1). After growing on potting mix for two weeks, focal individuals were planted into the mesocosm bins containing a blend of potting mix and field-collected soil with 2 replicates per community across 5 mesocosms, totaling 10 replicates per community per genotype, for a total of 60 focal individuals grown. Communities and mesocosm conditions are described below.

- Mesocosm community species composition

Each mesocosm contained three bins of the following community types to assess the effect of competitors on focal individuals and vice versa: focal individuals + competing weeds, focal individuals only, and competing weeds only. Potato plants were found to be weak competitors with no significant effects on the weeds (see Supplemental Table S1 for details); therefore, only results comparing focal plants under competition and focal plants alone are shown in the main text. To test for the effect of growing with common competitors, focal individuals were planted alongside the following heterospecifics: *Cyperus esculentus* (yellow nutsedge), *Plantago major* (broadleaf plantain), *Portulaca oleracea* (common purslane), and *Taraxacum officinale* (dandelion). These species were chosen to represent a variety of growth habits (rosette, prostrate, and erect), life histories (annuals and perennials), status (native and invasive), and reproductive strategies (outcrossing, self-fertilizing, and asexual propagation). In addition, all species are known to commonly occur across the globe and are considered invasive species outside of their native ranges. These competitors were germinated on potting mix in the greenhouse and grown for four weeks before transplantation into communities. Three replicates per competitor were randomly assigned positions within community bins across mesocosms to minimize position effects. While the ordering of position was randomized, planting was adjusted to ensure each focal individual grew alongside all four competitor species. For focal-only communities, each focal individual was planted in the same order as the focal + competitor within each mesocosm, approximately 50 cm away from each other, as they would be planted alone in the field. Comparisons of focal + competitor and focal only allow quantification of the effect of competition on growth likely to be found in nature.

- Mesocosm 1 growth conditions

Mesocosms were constructed inside the North Greenhouse at the University of Tennessee. Planting began August 2021, and the experiment continued until November 2021. During this period, the greenhouse temperature ranged from 20 °C to 44 °C. To supplement the available light, LED grow lights (Fluence SPYDR 2x LED Grow Light, Fluence, Austin, TX, USA) were installed and set to a photoperiod of 16 h light/8 h dark. Apogee MQ-500 light meters were used to verify the amount of total irradiance and spectrum of available light within the mesocosms. HOBO^®^ data loggers (Onset^®^, Bourne, MA, USA) were used to monitor temperatures within the mesocosms.

Communities were grown with a 1:1 mix of field soil (collected at East Tennessee AgResearch and Education Center (ETREC), Knoxville, TN, USA) to potting mix (PRO-MIX BK25, Premier Tech Horticulture, Quebec, Canada) by volume. Previous studies showed a combination of field soil and potting mix yielded the highest biomass of *S. tuberosum* in the designed mesocosms (Kakeshpour and Harbison, unpublished). Furthermore, while this soil composition is not solely derived from the field, it is likely to resemble natural conditions around agricultural fields where regular tilling and fertilization prevents field soil from compacting. Plants were watered as needed for the first two weeks to allow communities to be established, and then were watered with approximately 5.5 L per mesocosm twice weekly to allow for minor flooding and drying as occurs in nature. Plants were treated for pests with insecticidal soap 24, 35, and 45 days after planting. Each mesocosm was fertilized with Peters 20-20-20 fertilizer (J.R. Peters Inc., Allentown, PA, USA) after planting and monthly throughout the experiment.

- Mesocosm trait measurements

After senescence of focal individuals, harvest measurements of fresh aboveground biomass, number of shoots, stem mass, and number and mass of tubers were recorded for each focal individual. In Experiment 2 mesocosms, each month relative herbivory was scored as the ratio of leaves demonstrating insect damage out of the total number of leaves. Flower number was recorded, but no flowers set fruit for any genotype in any experiment; there was no significant difference in flowering time or number of flowers in either genotype or treatment.

#### 4.7.2. Mesocosm 2: Competition and Nutrient Availability Manipulation

- Plant propagation

Plants were generated from tubers collected from control plants in Experiment 1 (heat/photoperiod). Harvested tubers were stored at 4 °C for approximately 6 months, then placed in 10 cm pots filled with potting mix. Individuals were transplanted into mesocosms 3 weeks after sprouting.

- Mesocosm 2 communities

Mesocosm 2 communities were planted as either focal individuals (potato) only or focal individuals with heterospecific competitors. These communities were planted in the manner described in the previous mesocosm experiment. Each community type was exposed to either low (no supplemental fertilizer after initial planting) or high (fertilizer at levels to replicate average fertilizer runoff for this soil type applied weekly) nutrient conditions to evaluate whether nutrient availability affects response to competition across genotypes. Each community x nutrient treatment mesocosm was replicated six times for a total of 24 mesocosms.

- Mesocosm 2 growth conditions

Mesocosm 2 was planted in winter, beginning in January 2022. While supplemental light was maintained at the same 16 h light/8 h dark diurnal cycle of Mesocosm 1, the ambient photoperiod was reduced for these winter months. In addition, the temperature of the greenhouse was cooler, ranging from 20 °C to 29 °C. Mesocosms were grown until the plants senesced in April 2022, at which time the harvest measurements described in Experiment 2 were recorded.

### 4.8. Statistical analysis

Statistical analyses were completed using R v. 4.0.0 (R Core Team 2021). To test for the main effects of treatments, genotype and their interactions were regressed on each trait separately in generalized linear models by using the “glm” function in the stats package. Continuous traits such as aboveground biomass and tuber mass were calculated using Gaussian distribution, while discrete traits such as number of shoots and tubers were calculated using Poisson distribution. We used Type III analysis of variance to test for significance in all experiments.

Dunnett’s test was used to test for differences between control and multiple treatment groups or multiple transgenic lines to wildtype in the DescTools package. Effect sizes (pairwise differences) for each treatment within each genotype were calculated using the function “pairwise” in the emmeans package with Tukey’s method to adjust significance thresholds for multiple comparisons [89]. We used the Holm procedure to adjust *p*-values for multiple comparisons.

## Figures and Tables

**Table 1:** Abiotic stress ANOVA. Analysis of variance based on a generalized linear model to test for the effect of transgene, heat, and photoperiod on traits at harvest (Experiment 1). Transplanted individuals were grown under control, heat, and short photoperiod conditions until senescence. Likelihood ratio chi-squares for effect of transgene (‘Genotype’) and heat/photoperiod (‘Treatment’) are listed for aboveground traits (a) of number of nodes, plant height, and shoot fresh weight at harvest and belowground traits (b) of the number of tubers and tuber fresh weight. Significant chi-square values are bolded: *** *p* < 0.001 and * *p* < 0.05.

(a)				
**Fixed Effect**	**Df**	**Number of Nodes**	**Height**	**Shoot Fresh Weight**
		**LR ChiSq**	**LR ChiSq**	**LR ChiSq**
Genotype	10	11.626	96.44 ***	50.773 ***
Treatment	2	102.861 ***	598.55 ***	69.38 ***
Genotype × Treatment	20	13.428 ***	61.95 ***	40.574 *
(b)				
**Fixed Effect**	**Df**	**Number of Tubers**	**Tuber Fresh Weight**	
		**LR ChiSq**	**LR ChiSq**	
Genotype	10	5.5162	67.035 ***	
Treatment	2	0.7014	225.176 ***	
Genotype × Treatment	20	19.3275	64.806 ***	

**Table 2:** ANOVA based on GLM to test for the effect of transgene and competition on traits at harvest (Experiment 2). Transgenic line *StSP3D::OtsB* 10 and wildtype individuals were in competitive and non-competitive community mesocosms in the greenhouse during the summer. Likelihood ratio chi-squares for effect of transgene (‘Geno’) and community type (‘Competition’) are listed for number of shoots, fresh aboveground biomass, number of tubers, and fresh tuber mass. Df for each LR Chisq is 1. Significant chi-square values are bolded: *** *p* < 0.001, and * *p* < 0.05.

	Stem Number		Stem Mass		Biomass		Tuber Number		Tuber Mass	
Fixed effect	LR Chisq		LR Chisq		LR Chisq		LR Chisq		LR Chisq	
Geno	12.41	***	2.72		0.01		5.61	*	0.22	
Competition	21.37	***	0.98		29.63	***	38.24	***	3.95	*
Geno x Competition	2.1		4.31	*	1.74		0.5		0.91	

**Table 3:** ANOVA based on GLM to test for the effect of transgene, competition, and nutrient availability on traits at harvest (Experiment 4). Transgenic line *StSP3D::OtsB* 10 and wildtype individuals were grown in high and low nutrient regimes in competitive and non-competitive community mesocosms in the greenhouse during the winter. Likelihood ratio chi-squares for effects of transgene (‘Geno’), community-type (‘Competition’), and nutrient regime (‘Nutrient’) are listed for the number of shoots, fresh aboveground biomass, number of tubers, and fresh tuber mass. Df for each LR Chisq is 1. Significant chi-square values are bolded: *** *p* < 0.001, ** *p* < 0.01, and * *p* < 0.05.

	Number of Shoots	Aboveground Biomass	Number of Tubers	Tuber Mass
Fixed effect	LR Chisq	LR Chisq	LR Chisq	LR Chisq
Geno	0.18	1.32	2.77	0.09
Competition	51.63 ***	47.86 ***	4.20 *	2.14
Nutrient	0.43	0.48	49.40 ***	19.02 ***
Geno × competition	0.03	3.02	0.2	0.02
Geno × nutrient	6.10 *	9.13 **	5.45 *	1.46
Competition × nutrient	1	2.33	0.55	1.49
Geno x competition × nutrient	0.4	2.68	1.77	0.23

**Table 4:** Estimated marginal means for pairwise comparisons of effects of nutrient availability on mesocosm 2 (Experiment 3) harvest traits, based on GLM. Each pairwise comparison for high–low nutrient treatment for wildtype (‘WT’) and transgenic line *StSP3D::OtsB* within competitive (‘Neighbors’) and non-competitive (‘Alone’) mesocosms. Df is 1 for all estimated marginal means. Significant estimates are bolded: * *p* < 0.05 and + *p* = 0.05.

Trait	Geno	Community	Estimate (SE)		
Number of shoots	OtsB	Alone	−0.09	(0.19)	
Number of shoots	OtsB	Neighbors	−0.49	(0.29)	
Number of shoots	WT	Alone	0.36	(0.18)	+
Number of shoots	WT	Neighbors	0.27	(0.28)	
Biomass	OtsB	Alone	−32.05	(23.70)	
Biomass	OtsB	Neighbors	−26.48	(23.70)	
Biomass	WT	Alone	74.36	(21.94)	*
Biomass	WT	Neighbors	5.10	(21.94)	
Number of tubers	OtsB	Alone	−1.61	(0.49)	*
Number of tubers	OtsB	Neighbors	−3.14	(1.02)	*
Number of tubers	WT	Alone	−0.97	(0.31)	*
Number of tubers	WT	Neighbors	−0.98	(0.39)	*
Tuber mass	OtsB	Alone	−28.78	(9.41)	*
Tuber mass	OtsB	Neighbors	−22.45	(9.41)	*
Tuber mass	WT	Alone	−22.19	(8.71)	*
Tuber mass	WT	Neighbors	−7.13	(8.71)	

**Table 5:** Gene abbreviations.

Gene Name	Abbreviation
*Catalase*	*CAT*
*Elongation factor 1 alpha*	*EFI*
*Heat shock protein 30*	*HSP30*
*OtsB/trehalose-6-phosphate phosphatase*	*OtsB*
*StSP3D*	*3D*
*StSP6A*	*6A*
*60S ribosomal protein L8*	*L8*

## Data Availability

Data will be publicly available after publication on Figshare.

## Supplementary Materials

The following supporting information can be downloaded at: https://www.mdpi.com/article/10.3390/plants12193394/s1, Description of electrolyte leakage assay; Table S1. Results of an analysis of variance (ANOVA) test based on GLM (generalized linear model) for the effect of focal potato plants on weedy competitors; Table S2. Estimated marginal means for pairwise comparisons to test for effects of heat and photoperiod on phenotypes and yield of potato, based on GLM; Table S3. T-ratios for the estimated marginal means for pairwise comparisons, based on GLM, to test for effects competition on phenotypes and yield of potato in mesocosm experiments; Table S4. List of primers used for cloning and/or confirmation of transgenic lines or qRT-PCR of experimental samples to determine relative gene expression; Figure S1. Southern blot and PCR analysis of transgenic plant callus; Figure S2. Reaction norms of wildtype and transgenic lines in response to heat and photoperiod; Figure S3. Reaction norms of wildtype and transgenic plants in response to competition from mesocosm 1; Figure S4. Reaction norms of wildtype and transgenic plants in response to competition under high and low nutrient availability from mesocosm 2; Figure S5. Results of electrolyte leakage assay for wildtype and transgenic lines.
