# Supplementary material for: Heterologous Expression of OtsB Increases Tuber Yield and Phenotypic Stability in Potato under Both Abiotic and Biotic Stresses
Source: Plants (Basel). 2023 Sep 26;12(19):3394. doi: 10.3390/plants12193394 (PMC10574632; doi:10.3390/plants12193394)
Supplement: Supplementary file 1 [file plants-12-03394-s001.zip › plants-2586970-supplementary.pdf]

SUPPLEMENT

Electrolyte Leakage Assay

An electrolyte leakage assay was performed to measure the effect of heat on membrane injury in wildtype and transgenic lines. Samples were collected from potato plants grown in growth chambers under control conditions (16/8 h day/night photoperiod and 21/17 °C day/night temperatures) after growing in pots for about 3 weeks. A total of eight 10 mm leaf discs from four leaves (two per leaf) per plant were collected and placed into two Falcon tubes. Each tube contained four leaf disks, one from each leaf. Samples were soaked in distilled water for 30 min and rinsed gently. Leaf disks were then immersed in 10 mL distilled water in the same tubes. Half of the samples were incubated at 22 °C (control treatment) and the other half at 47 °C (heat treatment), with gentle shaking for 4 h. Samples were cooled to room temperature and their conductivity was measured using conductivity meter (Makarova et al., 2018).

Other studies suggest that trehalose acts as a protectant agains abiotic stress largely by stabilizing membranes. However, when analyzing results from an electrolyte leakage assay, we find no significant difference between the any transgenic line and wildtype (Figure S4), suggesting that the difference in phenotypes between genotypes is not mediated by changes in the cell membrane. This assay was performed from tissue samples collected from control plants, which were not exposed to heat stress. It is possible that trehalose accumulation is increased in transgenic plants when exposed to stress, but since we did not collect tissue samples for the electrolyte leakage assay from heat stressed plants, we cannot differentiate between these hypotheses. Further experiments on heat stress and cell membrane properties should be completed to determine whether the protective abilities demonstrated here are driven by alterations in cell permeability and structure.

Tables

Table S1. Results of an analysis of variance (ANOVA) test based on GLM (generalized linear model) for the effect of focal potato plants on weedy competitors. All mesocosms consisted of 4 individuals of each weed species: *Cyperus esculentus* (yellow nutsedge), *Plantago major* (broadleaf plantain), *Portulaca oleracea* (common purslane), and *Taraxacum officinale* (dandelion). Communities with plants were grown in mesocosm 1 either with four individual potato plants (two transgenic and two wildtype) or with competitors only; the competitors to replace focal potatoes in the competitors-only communities were chosen at random. These competitors were germinated on soil in the greenhouse and grown for four weeks before transplantation into communities. Three replicates per competitor were randomly assigned positions within community bins across mesocosms to minimize position effects, with each species occupying the same position in focal + competitors and competitor-only bins within each mesocosm. While the ordering of position was randomized, planting was adjusted to ensure each focal individual grew alongside all four competitor species. For competitor only bins, the position of each focal individual in focal + competitor was replaced with one of the competitors chosen at random. Comparisons of focal + competitor and competitor only quantify whether the focal individuals exert competitive pressure on these representations of communities likely to be found in nature. Based on the results below, potato were weak competitors with no effect on

weedy neighbors, as there was no significant effect of community or species by community interaction. Significant LR Chisq values are bolded with *** P < 0.0001.

	Biomass			Seed mass		
Fixed effect	*Df*	*LRChisq*		*Df*	*LR Chisq*	
Community	1	0.43		1	2.43	
Species	3	**145.24**	***	1	**83.30**	***
Community x Species	3	0.71		1	0.22	

Table S2. Estimated marginal means for pairwise comparisons to test for effects of heat and photoperiod on phenotypes and yield of potato, based on GLM. Each genotype is listed with the associated Z-ratio for each estimated marginal means for the effect of heat (heat effect) and photoperiod (SD effect. Significant values are bolded with *** P < 0.0001, **P < 0.001, and *P < 0.05.

Trait	Genotype	Heat effect		SD effect	
Height	WT	**4.38**	***	**-2.72**	*
	3D_1	**8.55**	***	-1.75	
	3D_10	0.26		**-5.48**	***
	3D_3	**7.80**	***	-1.58	
	3D_5	**4.56**	***	**-3.81**	***
	3D_6	**5.13**	***	**-2.94**	*
	6A_1	**4.96**	***	**-2.60**	*
	6A_4	**3.59**	***	-2.33	
	6A_5	**3.14**	***	-1.21	
	6A_7	**6.75**	***	-1.40	
	6A_8	**2.67**	*	-1.36	
Shoot fresh weight	WT	-1.43		-1.56	
	3D_1	**-3.02**	*	**-2.46**	*
	3D_10	**-3.98**	***	**-2.59**	*
	3D_3	-1.17		0.60	
	3D_5	**-2.64**	*	-0.96	
	3D_6	**-3.30**	***	0.23	
	6A_1	-0.58		-0.11	
	6A_4	**-2.80**	*	-0.56	
	6A_5	-2.28		2.37	
	6A_7	-2.02		1.82	
	6A_8	-1.28		1.67	
Tuber fresh weight	WT	**-10.84**	***	**4.29**	***
	3D_1	**-4.79**	***	0.56	

	3D_10	**-2.99**	*	1.87	
	3D_3	**-5.07**	***	**3.66**	***
	3D_5	**-4.84**	***	2.26	
	3D_6	**-2.85**	*	1.89	
	6A_1	**-3.10**	*	1.54	
	6A_4	**-4.68**	***	1.17	
	6A_5	**-3.70**	***	**3.30**	***
	6A_7	**-4.17**	***	0.66	
	6A_8	-1.73		2.10	
Number of nodes	WT	0.38		-1.07	
	3D_1	**3.07**	*	-1.02	
	3D_10	0.47		**-2.78**	*
	3D_3	2.35		-0.85	
	3D_5	2.02		-1.87	
	3D_6	1.67		-2.05	
	6A_1	1.65		-0.83	
	6A_4	1.24		-0.42	
	6A_5	0.81		-1.86	
	6A_7	**2.80**	*	-1.60	
	6A_8	0.76		-1.49	
Number of tubers	WT	-2.33		-0.71	
	3D_1	1.21		0.38	
	3D_10	-1.49		0.16	
	3D_3	-0.81		0.71	
	3D_5	0.00		-0.56	
	3D_6	0.17		0.49	
	6A_1	-0.99		-0.99	
	6A_4	0.23		0.00	
	6A_5	-0.47		0.22	
	6A_7	1.55		0.00	
	6A_8	0.26		0.50	

Table S3. T-ratios for the estimated marginal means for pairwise comparisons, based on the GLM, to test for effects competition on phenotypes and yield of potato in mesocosm experiments. The EMM +/- SE are shown in Figs. 3 and 4. In mesocosm 1, the mesocosms were grown with and without weedy competitors in the greenhouse during the summer months, resulting in hot conditions. In mesocosm 2, the mesocosms were grown with and without competitors under high and low nutrient availability during the winter months, resulting in cooler conditions. Both Experiments used supplemental grow lights to maintain a long-day (16hr) photoperiod. Wildtype is denoted as “Wt”, and the transgenic *StSP3D::OtsB* Line 10 is denoted as “OtsB”. Each genotype and mesocosm is listed with T-ratio associated with the estimated marginal means of competition in mesocosm 1 (Comp effect) and low nutrient (Comp effect Low-N) and high (Comp effect High-H) in mesocosm 2. Significant estimated marginal means are bolded with *** P < 0.0001, **P < 0.01, and *P < 0.05.

Trait	***Geno***	***df***	***Comp effect T-ratio***	***Comp effect Low-N T-ratio***	***Comp effect High-N T-ratio***
Biomass	Wt	24	**-5.43 *** **	**-5.89 *** **	**-2.79 ** **
	OtsB	24	**-1.65 ** **	**-2.26 * **	**-2.49 * **
Stem number	Wt	14	**-3.32 * **	**-3.17 ** **	**-3.86 ** **
	OtsB	14	-2.28	**-2.73 ** **	**-3.85 ** **
Stem mass	Wt	14	**-1.12 * **	N/A	
	OtsB	14	0.64		
Tuber number	Wt	24	**-2.28 * **	-1.65	-1.03
	OtsB	24	1.12	-0.29	-1.47
Tuber mass	Wt	24	0.64	0.05	**-1.81 * **
	OtsB	24	**1.32 * **	0.35	-1.04

Table S4. List of primers. The following primers were used for cloning and/or confirmation of transgenic lines or qRT-PCR of experimental samples to determine relative gene expression.

**Gene name**	**Forward primer**	**Reverse primer**
Catalase	TGGAAGCCAACTTGTGGTGT	ACTGGGATCAACGGCAAGAG
Elongation factor 1 alpha	GATGGTCAGACACGTGAACA	CCTTGGAGTACTTGGGGGTG
Heat shock protein 30	ATATCCGGGGATCGAGCAGA	TGTGTCAAGGAGGTCAGAGGA
OtsB (total expression)	CCGTTAACCGAAACCCCTGA	ATCACTTGCGGTTGCCAGTA
OtsB (transgene confirmation)	ATGACAGAACCGTTAACCGAAACCC	TTAGATACTACGACTAAACGACTCATAGTCATCACT
OtsB_BbsI	GATCGAAGACATAATGACAGAACCGTTAACCGA	GATCGAAGACTTAAGCTTAGATACTACGACTAAACGACTCATAGT

Phenylalanine ammonia lyase	TCGAGGACGAATTGAAGGCAA	CACCTGTCAACAGTTCTGATCC
SP3D	GGACCCAGATGCTCCAAGTC	CTTGCCAAAACTTGAACCTG
SP6A	GACGATCTTCGCAACTTTTACA	CCTCAAGTTAGGGTCGCTTG
SP3D_BbsI	ACTGGAAGACTAGGAGCTGGTTAAGTCTCTGAGATGAAGTG	GATCGAAGACGCCATTCTCTAGGCAAGATGATGGTTCA
SP6A_BbsI	ACTGGAAGACTAGGAGCCCTTAGATCGTATGCATTGA	GATCGAAGACGCCATTATGGATCAACTCTAGGCAGGAT
60S ribosomal protein L8	TGGTAATGTGTTGCCGCTTA	GGCGTAATCACCAGAACACC

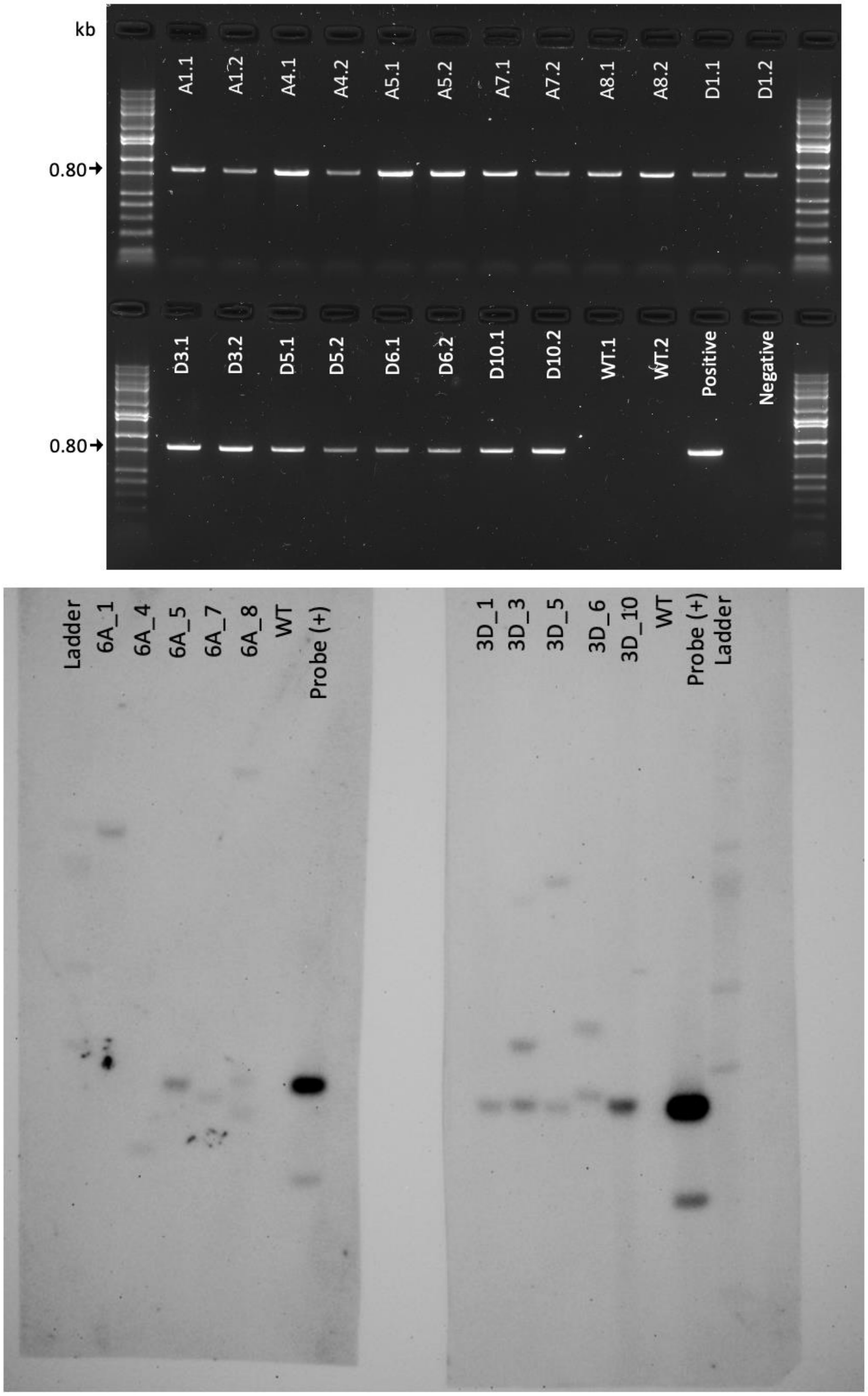

Figure S1. PCR confirmation and Southern blot verification of transgenic plant callus. After calli were down-selected on antibiotic media, plants were grown on soil for 4 weeks in duplicate. DNA was extracted from leaf tissue to confirm presence of transgene using PCR of the OtsB gene (top). Copy number of transgenes were estimated by the Southern blot analysis on two separate blots: Blot 1 (bottom left) and Blot 2 (bottom right).

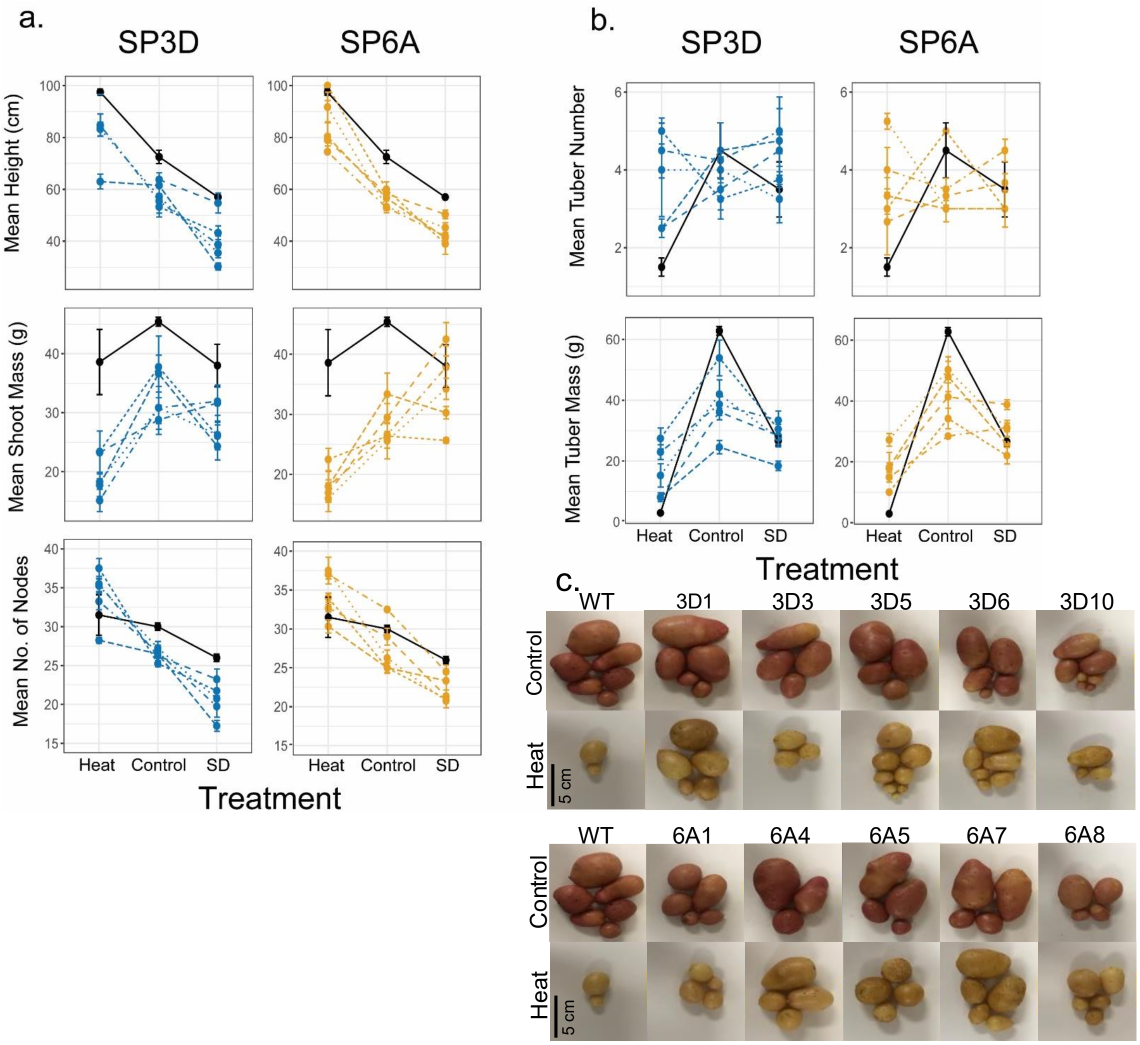

Figure S2. Reaction norms of wildtype and transgenic lines in response to heat and photoperiod. The mean and standard error of the aboveground traits (a) of height, shoot fresh weight, and number of nodes and the belowground traits (b) of tuber number and mass are plotted. Line type demonstrates different genotypes (independent lines) while line color represents wildtype in black, StSP3D constructs in blue, and StSP6A constructs in gold. Treatment is shown on the x-axis, with control measurements in the center of heat and short day treatments, respectively. Representative images of tuber harvest in wild type and each line are shown (c).

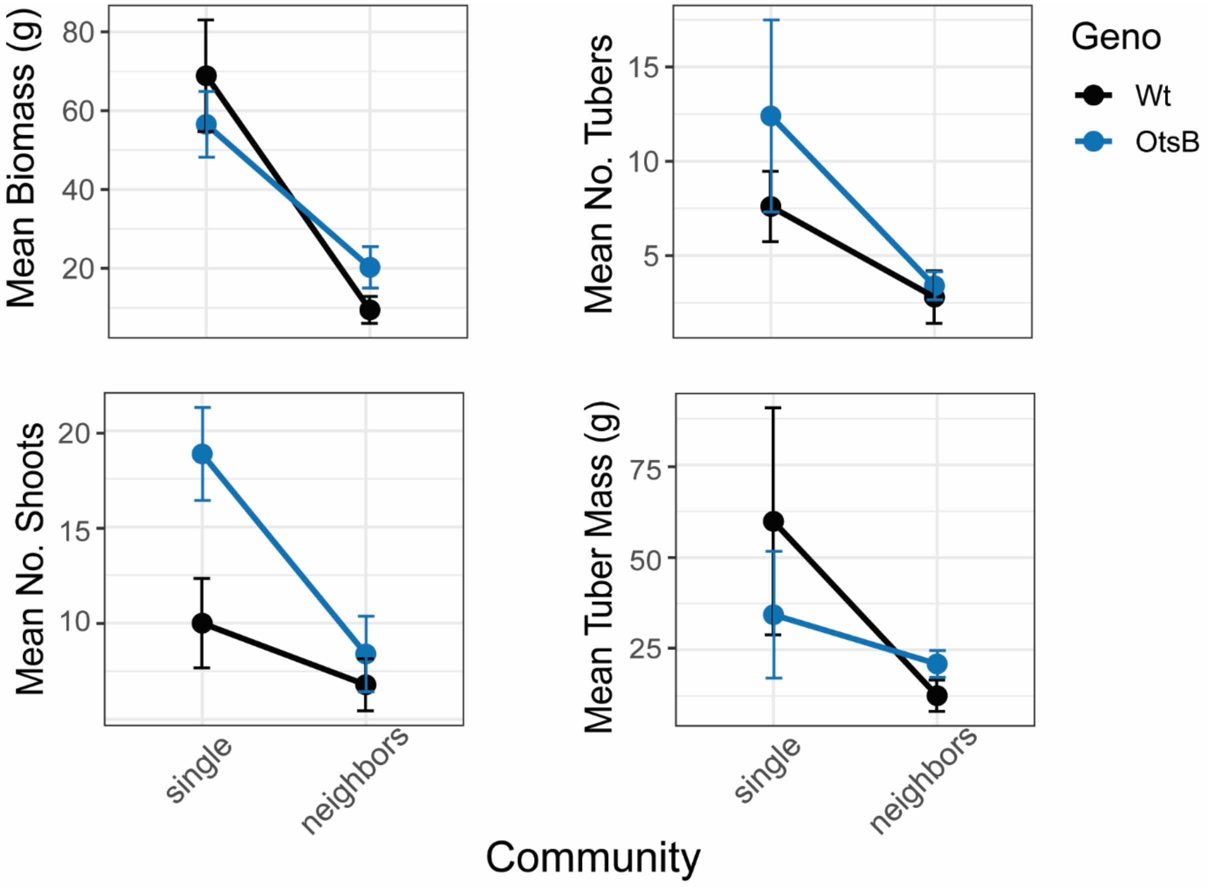

Figure S3. Reaction norms of wildtype and transgenic plants in response to competition from mesocosm 1. The mean and standard error of the aboveground traits (left) of biomass and number of shoots and the belowground traits (right) of tuber number and mass are plotted. Genotype is demonstrated by line color, with wildtype in black and StSP3D Line 10 in blue. Community type x-axis, with those grown alone (single) on the left and those grown with competitors (neighbors) on the right.

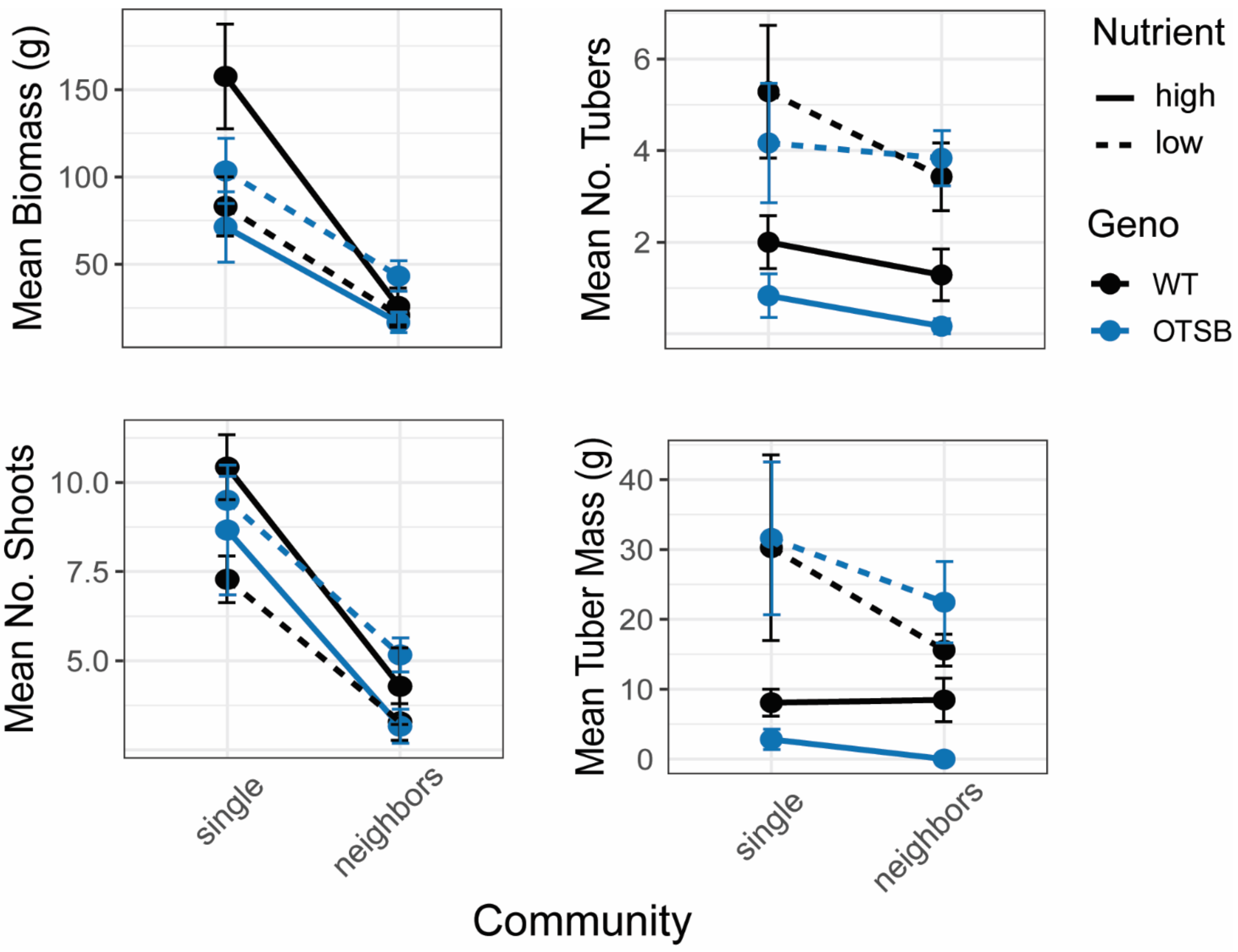

Figure S4. Reaction norms of wildtype (WT) and transgenic plants in response to competition under high and low nutrient availability from mesocosm 2. The mean and standard error of the aboveground traits (left) of biomass and number of shoots and the belowground traits (right) of tuber number and mass are plotted. Line type demonstrates different nutrient regimes with high nutrient availability in solid lines and low nutrient availability in dashed lines. Genotype is demonstrated by line color, with wildtype in black and StSP3D Line 10 in blue. Community type x-axis, with those grown alone (single) on the left and those grown with competitors (neighbors) on the right.

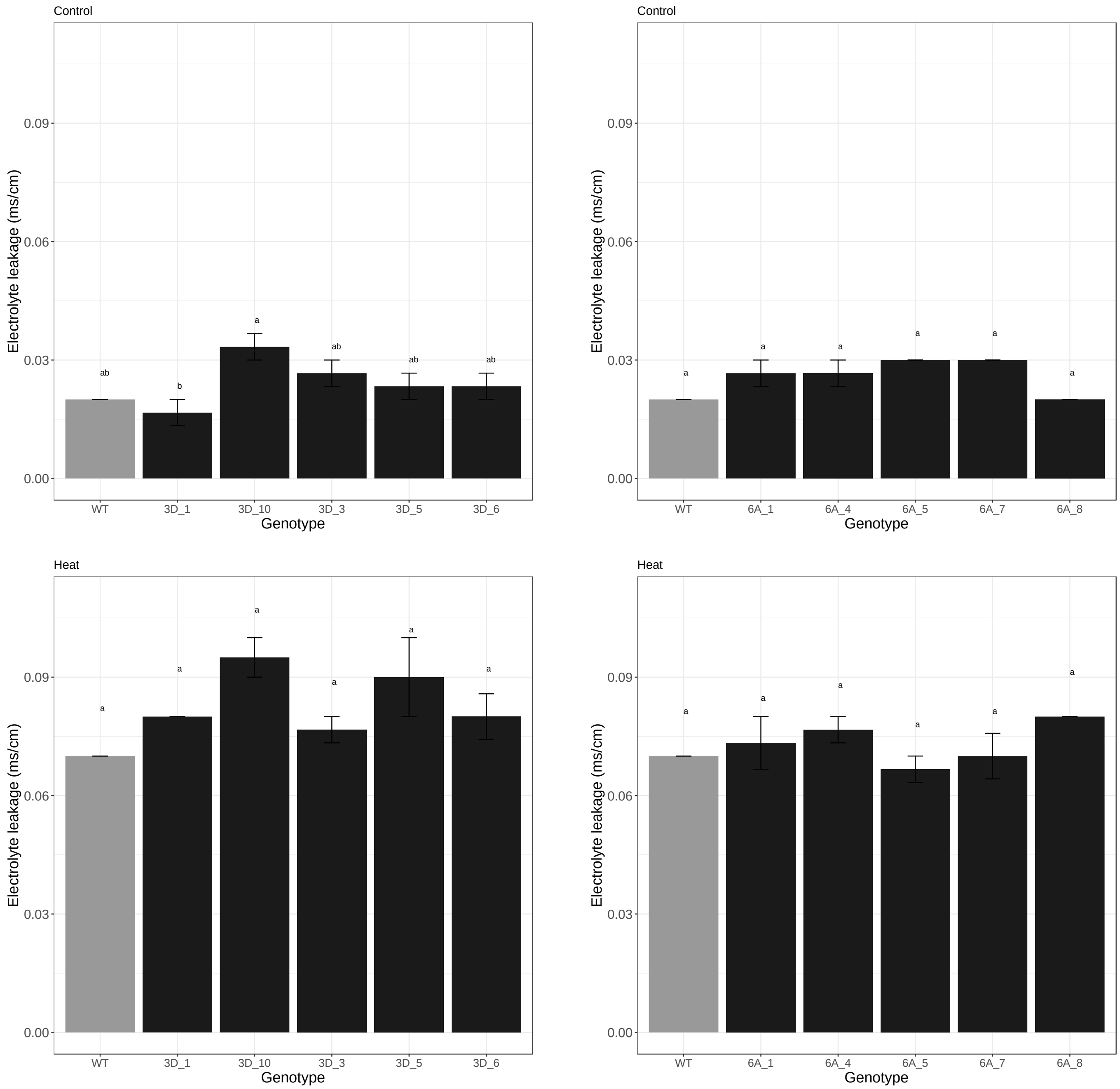

Figure S5. Electrolyte leakage assay for wildtype and transgenic lines. OtsB::SP3D (3D), OtsB::SP6A (6A).
